# Supporting Older Adults and Caregivers Through Integrative Service Learning During COVID

**DOI:** 10.1093/geroni/igaa057.3503

**Published:** 2020-12-16

**Authors:** Keith Chan, Sarah LaFave, Maggie Ratnayake, Christina Marsack-Topolewski, Jillian Graves, Diane Fenske, Shay Lukas

**Affiliations:** 1 Hunter College, City University of New York, New York, New York, United States; 2 Lori’s Hands, Inc, Newark, Delaware, United States; 3 Lori’s Hands, Inc., Newark, Delaware, United States; 4 Eastern Michigan University, Ypsilanti, Michigan, United States; 5 University of Delaware, Newark, Delaware, United States

## Abstract

There is a growing population of older adults who are living longer and acquiring chronic illness and disabilities, making it difficult for them to complete everyday activities and age in place. More than 2 million of these older adults are homebound and 5 million need help leaving their homes. They experience social isolation, food insecurity, and lack of connection to community resources which has intensified since the pandemic. Integrative service learning models can provide home-based support to older adults while offering valuable, hands-on learning experiences for students. This study examined findings for a community-based program which trained university students to provide practical home-based support for older adults and their caregivers. Data was collected for 109 older adults who were connected with student trainees. Students provided services with groceries, companionship, and help accessing needed services. Findings from t-test results using the UCLA Loneliness Scale indicated that older adults reported less loneliness after engagement with students (mean difference = 6.15, t = 3.14, df = 82, p < 0.01). Qualitative process data suggested that older adults benefited from services and a connection to their assigned students prior to and during the pandemic. Student trainees reported that the experience enriched their learning and reaffirmed their commitment to working with older adults. Community-based service learning can address home-based needs of older adults and their caregivers and enhance learning opportunities for students. Policies and practice can support a pipeline of geriatric health professionals through innovative service learning models to benefit older adults, caregivers and students.

